# Supplementary material for: INSurVeyor: improving insertion calling from short read sequencing data
Source: Nat Commun. 2023 Jun 5;14:3243. doi: 10.1038/s41467-023-38870-2 (PMC10241795; doi:10.1038/s41467-023-38870-2)
Supplement: Supplementary file 1 — Supplementary Information [file 41467_2023_38870_MOESM1_ESM.pdf]

Supplementary Information

INSurVeyor: improving insertion calling from short read sequencing data

Supplementary Methods

Detecting discordant pairs and clipped reads

INSurVeyor starts from a BAM or CRAM file and searches for two types of evidence used to detect insertions: discordant pairs and clipped reads. Discordant read pairs are pairs of reads that map inconsistently to the library preparation parameters. In particular, we are interested in pairs s.t. the two reads either map far apart (more than 100 kbp apart) or they map to different chromosomes. These pairs may indicate the presence of an insertion due to transposition. Furthermore, we are interested in pairs s.t. only one read is mapped, while the other is unmapped. These pairs may indicate the presence of a novel insertion.

Clipped reads are reads that are only partially mapped to a reference location. This may happen because the read spans an insertion breakpoint, i.e., part of the read is sequenced from the inserted sequence and the rest is sequenced from the flanking reference sequence. Therefore, clipped reads may be evidence of the presence of an insertion. Furthermore, they can be useful for identifying the precise breakpoints of the insertion.

In this step, INSurVeyor also estimates some parameters of the input library that will be useful in subsequent steps: the average read depth, a minimum and a maximum acceptable depths (computed as the 1st and 99th percentile of the read depth distribution of the library), and a maximum acceptable insert size $maxIS$ (computed as the average insert size plus 3 standard deviations).

Assembling the alternative allele

The second step of INSurVeyor assembles the alternative allele for each putative insertion. Three different modules target different types of insertions: transpositions (remapping module), novel insertions (local assembly module) and small insertions (consensus-overlap module). In this context, we refer to an insertion as “transposition” when a copy of the inserted sequence is present in the reference genome, and “novel insertion” when this is not the case.

Remapping module

Given a discordant pair supporting a transposition, one of the reads will map to the insertion site while the other will map to a copy of the inserted sequence. We call the former *stable read* and the latter *unstable read.* This is because

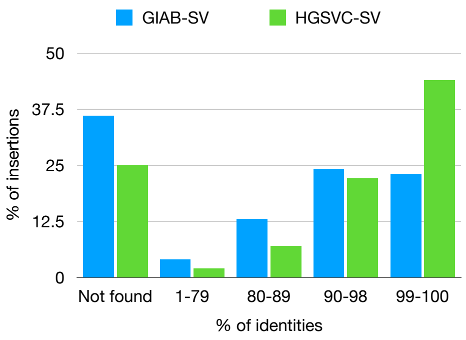

Supplementary Figure 1: **Inserted sequences are not accurately represented in the reference.** We aligned the inserted sequences of the GIAB-SV and HGSVC2 catalogues to the reference human genome using BLAST (query coverage $\geq 90\%$). For each sequence, we kept the hit with the highest bitscore. Then, we partitioned the hits based on the percentage of identical matches in the alignment, as reported by BLAST. Less than half of the insertions have an accurate representation in the reference ($\geq 99\%$ matches).

the inserted sequence is often present in many copies throughout the reference genome, and unstable reads can be mapped to multiple locations. In practice, we do not know which read is stable and which is unstable, so we use a simple heuristic: the read with the highest mapping quality is labelled as stable read. This fast and simple strategy was shown to be more effective than more involved strategies [1]. Special attention is given to pairs where both reads have the same mapping quality, and the two reads are labelled both stable and unstable. Pairs where both reads have low mapping quality (<20) are discarded.

Next, stable reads are clustered so that all of the reads belonging to the same stable cluster are on the same chromosome, on the same strand and no more than $maxIS$ bp from each other. Details for the clustering algorithm can be found in [1]. Clipped reads are partitioned into left-clipped (their left end is clipped) and right-clipped (right end is clipped). Then left-clipped (resp. right-clipped) reads are clustered so that their left (resp. right)-end coordinates are within 5 bp from each other. For each clipped cluster, a consensus sequence is built by casting a majority vote base by base. If a stable cluster on the positive (resp. negative) strand overlaps with a right (resp. left)-clipped cluster, the clipped cluster joins the stable cluster (Supplementary Figure 4a). A stable cluster is only allowed one clipped cluster, and if it overlaps many suitable clipped clusters, the largest (by number of reads) is chosen. The outcome of the clustering process is a set of positive clusters and a set of negative clusters.

Pairs of positive-negative clusters are created so that, for each pair, the right end of the positive cluster and the left end of the negative cluster are less than $maxIS$ bp apart, and each cluster only belongs to one pair. Each pair represents a potential insertion (Supplementary Figure 4b): the stable clusters define a tentative insertion site, and the unstable mates are fragments of the inserted sequence.

Given a pair of clusters, we want to find the region in the reference genome where the unstable reads align best overall. Testing the whole reference would be computationally extremely expensive, therefore we identify a set of *candidate*

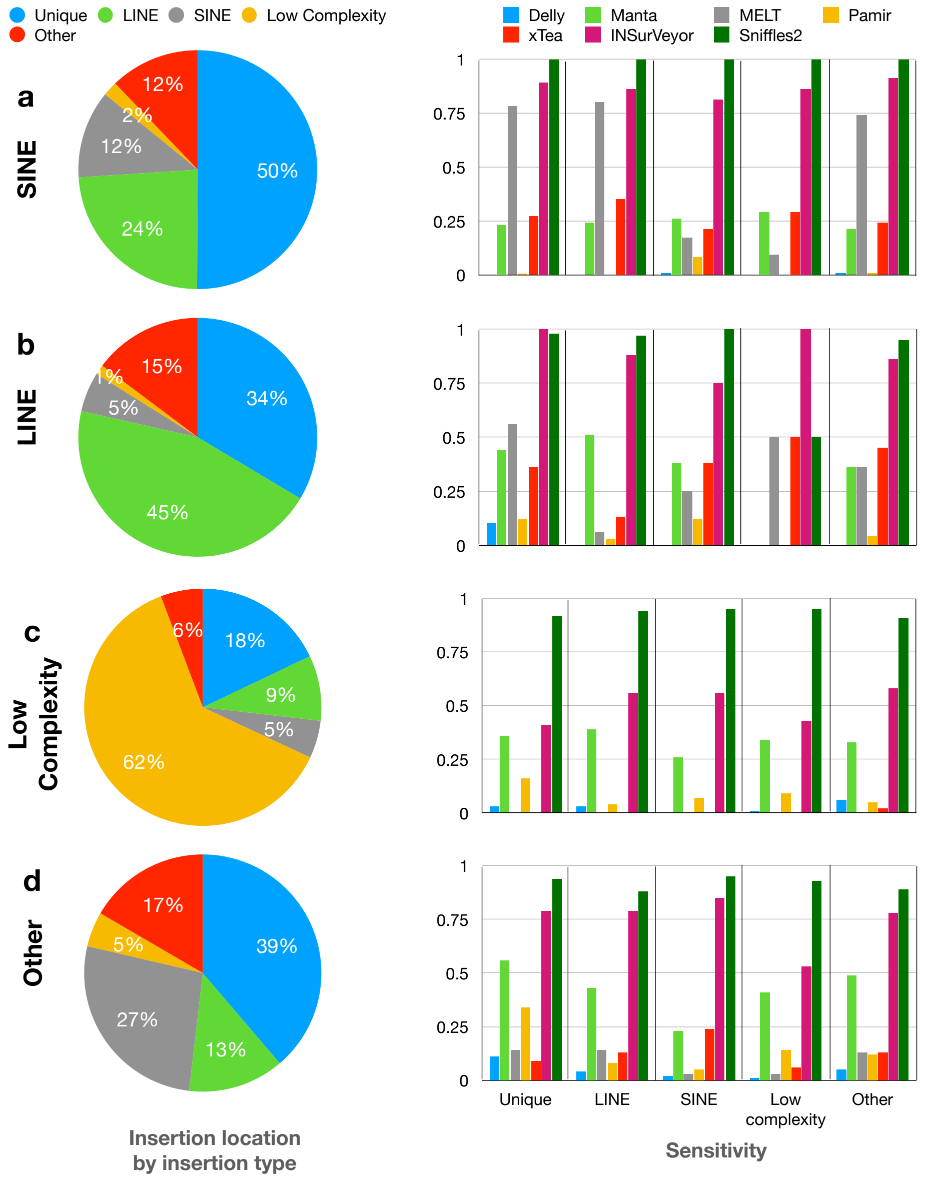

Supplementary Figure 2: **Performance of different callers stratified by the repeat content of the inserted sequence as well as the insertion site.** We break down the performance of the tested short reads methods, plus Sniffles2, according to the type of the inserted sequence and the insertion site. The benchmark insertions are partitioned into four types depending whether the inserted sequence is (a) SINE, (b) LINE, (c) low complexity or (d) other, i.e., none of the previous categories. SVA were too few to obtain meaningful statistics by themselves, so they were included in (d). For each category, the insertions are further sub-classified according to the RepeatMasker annotation of the insertion sites. The sensitivity of different tools was assessed for each sub-class. INSurVeyor performs better than the other short reads callers in every single class. As previously noted, long reads present a large advantage when detecting insertions of low complexity regions. In other categories, INSurVeyor was able to detect most insertions detected by Sniffles2.

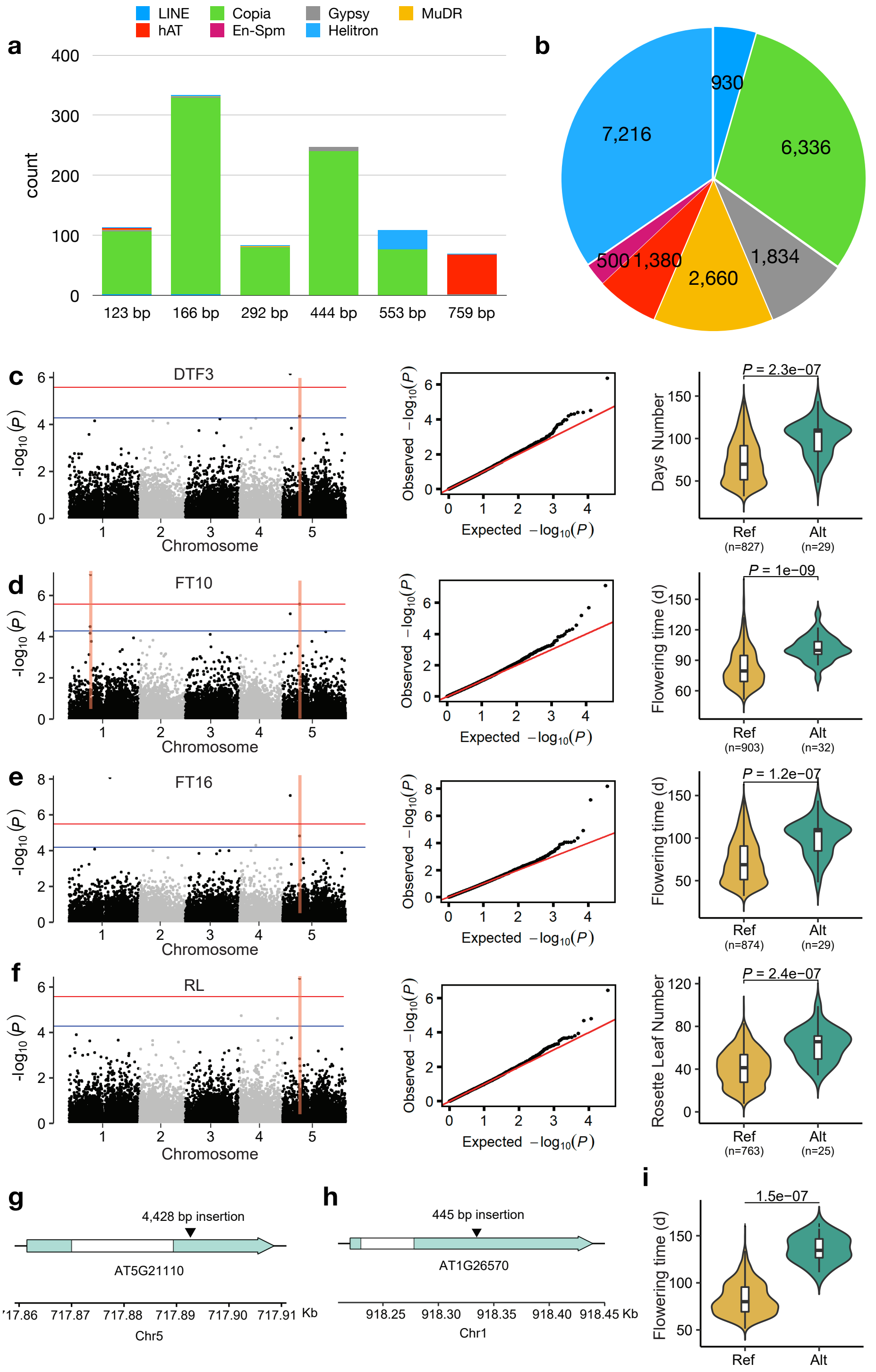

Supplementary Figure 3: **Additional properties of the catalogue of insertions called in 1,047 samples from the 1001 Genomes Project.** (a) Classification of TE insertions for each peak in the size distribution. (b) Number of TE insertions identified by INSurVeyor across different TE superfamilies. (c-f) A significant loci for days until first open flower (DTF3) (c), flowering time under 10°C (d), flowering time under 16°C (e), and rosette leaf number (RL) (f). (g) The 4,428 bp insertion inserted in the second exon of *AT5G21110*. (h) The 445 bp insertion inserted in the second exon of *AT1G26570*. (i) The 11 accessions with the 445 bp insertion flowered later than those without it. Boxplots in (c), (f), and (I) show median (inner line) and inner quartiles (box). Whiskers extend to the highest and lowest values no greater than 1.5 times the inner quartile range. P values were determined using two-tailed Student's t-tests.

regions to test. For each unstable end, the region surrounding it is a candidate region (size is user-defined, 10 kbp by default). We compute the score of a candidate region by performing a Smith-Waterman alignment between the region and each unstable read, and summing the alignment scores. We enforce additional constraints on the alignments: (a) all the unstable reads in a positive (resp. negative) strand must align on the same strand, and within $maxIS$ from each other; (b) unstable reads in a positive cluster and unstable reads in a negative cluster must align to different strands (Supplementary Figure 4c). The candidate region with the highest score is called the *best candidate region*.

Next, we aim at using the best candidate region to guide the assembly of the alternative allele. We build a draft of the alternative allele by joining the reference regions flanking the tentative insertion site with the best candidate region, and we realign all reads (both stable and unstable) to it (Supplementary Figure 4d). Reads that fully align to the draft sequence and overlap each other are merged into contigs, while reads that are clipped or align poorly are assembled separately into scaffolds (Supplementary Figure 4e). Lastly, contigs and scaffolds are assembled into the final alternative allele sequence (Supplementary Figure 4f).

If the alternative allele was assembled correctly, all reads supporting the insertion, i.e., clipped or belonging to a discordant pair, should fully align to the alternative allele (allowing for $\epsilon\%$ mismatches to account for sequencing errors, $\epsilon = 4$ by default). In practice, as previously mentioned, most inserted sequences do not have a copy in the reference, the algorithm may not find a good candidate region. This may cause the remapping module to fail. If more than 50% of the reads do not fully align to the alternative allele, or they have too many mismatches, we fall back to the local assembly module which uses de novo assembly.

Local assembly module

The local assembly module considers each positive-negative cluster that failed the remapping module one at a time, and aims at assembling its alternative allele de novo. The main idea is to find a subset of reads to assemble into the alternative allele and to find the most probable ordering from left to right by sequencing position on the alternative allele (Supplementary Figure 5a). After that, we consider the reads in that order and we extend the contig from left to right one read at the time.

In order to do so, we generate a directed graph where every read is represented as a vertex, and each edge between two vertices represents a relative ordering between the two corresponding reads. Given a read R, let $prefix(R, s)$ be the prefix of length s of R; similarly, $suffix(R, s)$ is the suffix of length s of R. For each pair of reads R_1, R_2, we find the largest S such that $suffix(R_1, S)$ matches $prefix(R_2, S)$ ($\epsilon\%$ mismatches allowed, gaps disallowed). If $S \geq minS$ ($minS = 15$ initially), an edge is drawn from R_1 to R_2 (Supplementary Figure 5b).

We enforce additional constraints on the graph. The reads are partitioned into positive stable, unstable and negative stable, and we can establish a partial ordering between them. By definition, as exemplified by Supplementary Figure 5a, positive stable reads must come before unstable reads, and unstable reads must come before negative stable reads, if considered from left to right.

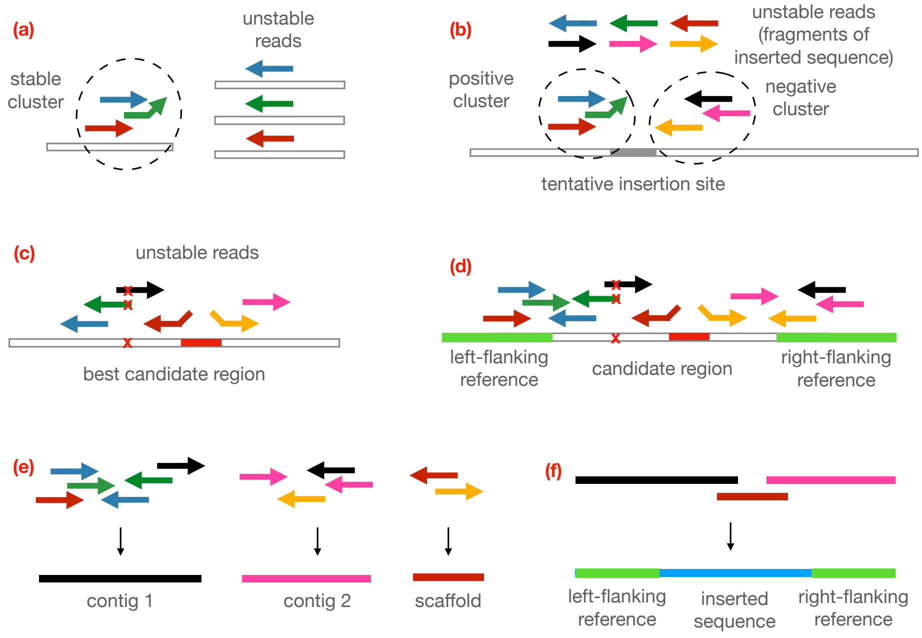

Supplementary Figure 4: Workflow of the remapping module. (a) Stable reads and clipped reads are clustered into stable clusters. Reads with the same colour belong to the same pair. (b) A stable cluster is called a positive (or negative) cluster if it aligns on the positive (or negative) strand of the reference genome. A nearby pair of a positive and a negative cluster defines a tentative insertion site. The corresponding unstable reads are potential fragments of the inserted sequence. (c) The unstable reads are aligned to several candidate regions, and the region where the reads align best is chosen as the best candidate region. However, the best candidate region may not be identical to the actual inserted sequence: in the case illustrated, the actual inserted region has a SNP and a deletion (in red) compared to the best reference region. (d) A draft sequence of the alternative allele is created, by joining the reference sequences flanking the insertion site and the best candidate region. All reads, both unstable and stable, are realigned to the draft of the alternative allele. (e) Reads that fully align to the draft sequence and overlap each other are merged into contigs. Reads that are clipped or align poorly are assembled separately into scaffolds. (f) Finally, contigs and scaffolds are assembled into the final alternative allele sequence.

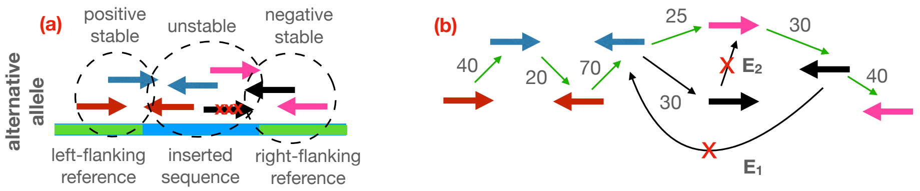

Supplementary Figure 5: **Workflow of the local assembly module.** It starts from the end of step (b) in Supplementary Figure 4, from a pair of a positive and a negative stable clusters. (a) The alternative allele to be assembled, and how the reads align to it. Note that, although the alternative allele is not known, the partial ordering positive stable → unstable → negative stable must hold. (b) A directed graph is built where each vertex represents a read. An edge is created from a read R_1 to a read R_2 if the suffix of R_1 and the prefix of R_2 match (ϵ% mismatches allowed), and the weight of the edge is proportional to the length of the match. (b) also shows the benefits of fixing the partial ordering positive stable → unstable → negative stable. The edge E_1 would create a cycle; however, an unstable read cannot follow a negative stable read, so the edge is removed. Furthermore, due to a noisy black read, E_2 was also disallowed, and greedily following the highest scoring edge would result in an incomplete assembly. However, by computing the heaviest path, INSurVeyor is still able to assemble the whole alternative allele.

Therefore, we do not allow edges from the unstable reads to the positive stable reads, and we do not allow edges from the negative stable reads to unstable and positive stable reads. This observation reduces the probability of incorrect orderings and cycles in the graph. A second constraint is that the graph must be acyclic, otherwise the same read(s) would be used multiple times in the assembly process. If the graph is cyclic, we progressively increase $minS$ by 10 and rebuild the graph until it is acyclic.

Finally, the heaviest path in the graph is found, representing the ordered subset of reads, which are then assembled into the alternative allele.

Consensus-overlap module

Both the remapping module and the assembly module may fail to predict very short insertions (shorter than the read length) due to a lack of discordant pairs. The gap is filled by the *consensus overlap module*, which discovers insertions only using clipped reads. The module uses the clipped consensuses produced by the remapping module (Supplementary Figure 6a), and uses them to predict insertions without relying on discordant pairs.

Right-clipped and left-clipped consensuses are paired so that (a) the end of the right-clipped cluster and the start of the left-clipped cluster are no more than 50 bp apart; (b) each cluster belongs to only one pair. For each pair, let r be the consensus sequence of the right-clipped cluster, and l be the consensus sequence of the left-clipped cluster. We find the largest integer S s.t. $suffix(r, S)$ matches $prefix(l, S)$ (ϵ% mismatches allowed, gaps disallowed). If $S \geq minS$ (15 by default), we join the two consensus into the alternative allele sequence (Supplementary Figure 6b).

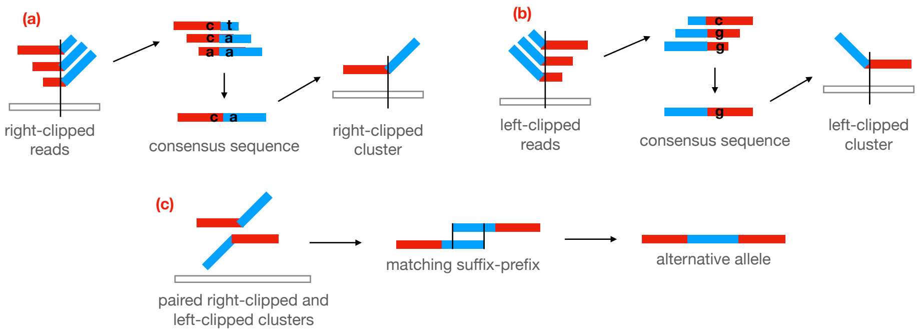

Supplementary Figure 6: **Workflow of the consensus-overlap module.** (a) Right-clipped reads are clustered so that their end coordinates are within 5 bp from each other. A consensus sequence is built for each cluster. (b) Similarly, left-clipped reads are clustered so that their start coordinates are within 5 bp from each other. (c) Right-clipped and left-clipped clusters are paired if their end and start coordinates, respectively, are within 50 bp from each other. If the suffix of the right-clipped cluster matches a prefix of the left-clipped cluster not shorter than 15 bp, we merge them into the alternative allele.

Filtering

INSurVeyor mainly uses two types of evidence to filter false positives: anomalous coverage of the insertion site and low support for the insertion.

Coverage filter : An approximate distribution of the read coverage of the sample is computed by sampling the coverage at one million randomly generated coordinates. Let $minCov$ (resp. $maxCov$) be an integer s.t. 1% of the values in the distribution are lower (resp. higher) than it. If the coverage of the stable regions is below $minCov$ or above $maxCov$, we consider it to be anomalous and we filter the insertion. Furthermore, we filter the insertion if more than $maxCov$ clipped reads support the insertion.

Low discordant pairs filter : For each $d \in [50..maxIS]$, let S_d be minimum number of discordant pairs that we expect to support an insertion of size d. We estimate S_d by randomly selecting a region of size d, and counting the number of read pairs s.t. one read is mapped within the region while the other is mapped outside of it. By repeating this procedure one million times, we obtain a D_d distribution with one million points. We select S_d so that it is greater than 1% of the points in D_d. Finally, for each predicted insertion I, let l be the length of its inserted sequence. If the number of discordant pairs supporting I is lower than S_l, we filter the insertion. This filter is not applied to the consensus-overlap module.

Positive-to-negative ratio filter : Let p be the number of pairs and clipped reads supporting the insertion, and let n be the number of pairs and clipped reads that do not support the insertion (i.e., non-discordant pairs that span the insertion site and reads that span the putative breakpoints but are not clipped). We compute the positive-to-negative ratio as $p/(p+n)$. The higher the value, the more confident we are that the insertion is real. By default, INSurVeyor filters

insertions with a positive-to-negative ratio lower than a threshold r (0.25 by default).

Supplementary Figures 7-10

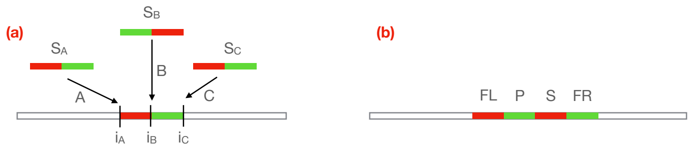

Supplementary Figure 7: **A tandem duplication can be represented as several equivalent insertions.** (a) A region (highlighted in red and green) is duplicated. This can be represented as an insertion in several ways: three examples (labelled A, B and C) are shown. S_t and i_t are the inserted sequence and the insertion site, respectively, of insertion t, $t \in \{A, B, C\}$. Although it is straightforward to see that A and C lead to a tandem duplication, this is not the case for B, because the inserted sequence is different from the duplicated reference sequence. However, it is easy to verify that all three insertions lead to the same result, shown in (b). In general, if we can partition the inserted sequence into two sequences P and S, and P matches the sequence FR immediately to the right of the insertion site, and S matches the sequence FL immediately to the left of the insertion site, the insertion is a duplication. This is because the resulting alternative allele will be $FL.P.S.FR$. Since $FL = S$ and $FR = P$, this is equivalent to $FL.FR.FL.FR$, which is a tandem duplication of the original sequence.

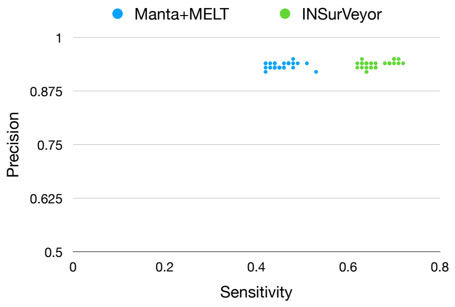

Supplementary Figure 8: **Performance of Manta+MELT and INSurVeyor for 34 samples in the HGSVC2 benchmark.** X-axis and Y-axis represent sensitivity and precision, respectively, and each dot is a sample. While the precision is similar, INSurVeyor has consistently higher sensitivity.

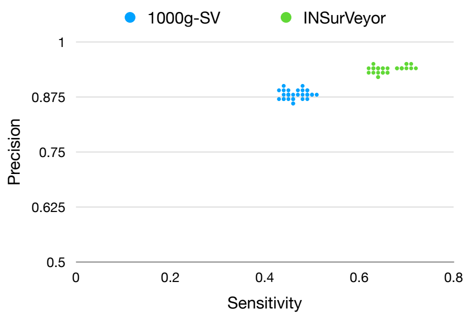

Supplementary Figure 9: **Performance of 1000g-SV and INSurVeyor for 34 samples in the HGSVC2 benchmark.** X-axis and Y-axis represent sensitivity and precision, respectively, and each dot is a sample. INSurVeyor has consistently higher sensitivity and precision.

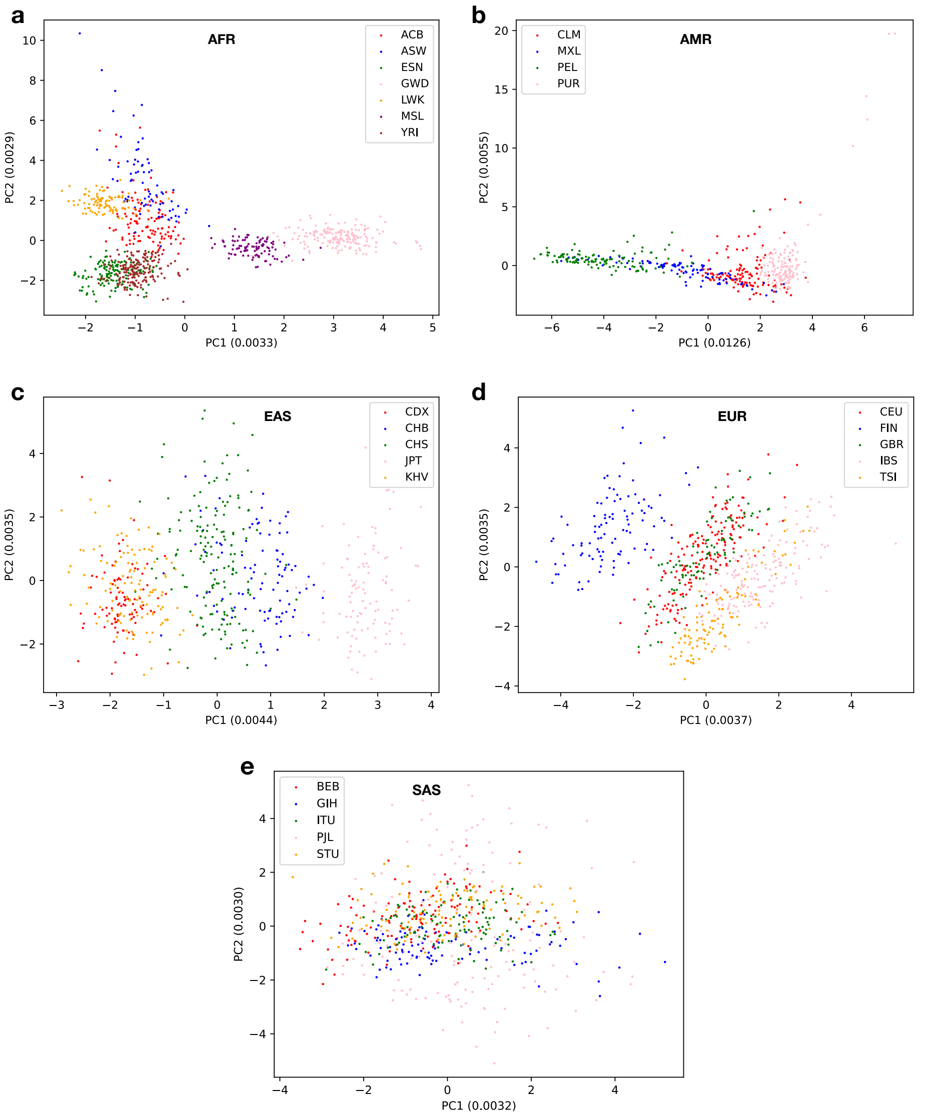

Supplementary Figure 10: **PCA based on our insertions catalogue is also able to segregate each superpopulation into subpopulations.** (a) Africans (AFR) appear to be the most diverse superpopulation, and the subpopulations are segregated very clearly. (b) Americans (AMR) subpopulations are segregated on PC1, while PC2 is dominated by heterogeneity among Puerto Ricans (PUR). (c) East Asians (EAS) form three major clusters: (1) Vietnamese and Dai Chinese, (2) Han Chinese with Southern Han Chinese and (3) Japanese. Similarly (d), Europeans (EUR) are also clustered into three clusters: (1) Finnish, (2) British and Utah residents with Northern and Western European ancestry and (3) Italians from Tuscany and Spanish. (e) South Asians (SAS) structure is much less obvious than other superpopulations.

Supplementary Tables

Gene	Pathology	Samples	Motif detected	Notes
RFC1	CANVAS	683	Several, most common ones are AAAAG, AAAGG, AAAGGG	Pathogenic motif AAGGG is found in 12 individuals
ATAXN10	SCA10	12	ATTCT	Expansion in the intermediate range (30-799) found in 12 individuals, all from Central and South America, where SCA10 is predominantly found
BEAN1 - TK2	SCA31	0	-	Expansion of motif TGGAA usually not found in healthy individuals
DAB1	SCA37	92	AAAAT	Pathogenic motif ATTTC not found
FXN	Friedreich's Ataxia	59	GAA	Very long expansions (>2kb) are pathogenic

Supplementary Table 1: Polymorphism found by INSurVeyor in 1000 genome samples for loci where STR expansions in intronic ALUs are known to be pathogenic.

Supplementary References

[1] Rajaby, R., Sung., W.K. Transurveyor: an improved database-free algorithm for finding non-reference transpositions in high-throughput sequencing data. *Nucleic Acids Res*, 46(20):e122 (2018)
