# Supplementary material for: INSurVeyor: improving insertion calling from short read sequencing data
Source: Nat Commun. 2023 Jun 5;14:3243. doi: 10.1038/s41467-023-38870-2 (PMC10241795; doi:10.1038/s41467-023-38870-2)
Supplement: Supplementary file 3 — Reporting Summary [file 41467_2023_38870_MOESM3_ESM.pdf]

nature portfolio

Corresponding author(s): Wing-Kin Sung

Last updated by author(s): Mar 6, 2023

Reporting Summary

Nature Portfolio wishes to improve the reproducibility of the work that we publish. This form provides structure for consistency and transparency in reporting. For further information on Nature Portfolio policies, see our Editorial Policies and the Editorial Policy Checklist.

Statistics

For all statistical analyses, confirm that the following items are present in the figure legend, table legend, main text, or Methods section.

n/a	Confirmed	
☐	☒	The exact sample size (*n*) for each experimental group/condition, given as a discrete number and unit of measurement
☒	☐	A statement on whether measurements were taken from distinct samples or whether the same sample was measured repeatedly
☐	☒	The statistical test(s) used AND whether they are one- or two-sided<br>*Only common tests should be described solely by name; describe more complex techniques in the Methods section.*
☒	☐	A description of all covariates tested
☒	☐	A description of any assumptions or corrections, such as tests of normality and adjustment for multiple comparisons
☐	☒	A full description of the statistical parameters including central tendency (e.g. means) or other basic estimates (e.g. regression coefficient) AND variation (e.g. standard deviation) or associated estimates of uncertainty (e.g. confidence intervals)
☐	☒	For null hypothesis testing, the test statistic (e.g. *F*, *t*, *r*) with confidence intervals, effect sizes, degrees of freedom and *P* value noted<br>*Give P values as exact values whenever suitable.*
☒	☐	For Bayesian analysis, information on the choice of priors and Markov chain Monte Carlo settings
☒	☐	For hierarchical and complex designs, identification of the appropriate level for tests and full reporting of outcomes
☒	☐	Estimates of effect sizes (e.g. Cohen's *d*, Pearson's *r*), indicating how they were calculated

Our web collection on statistics for biologists contains articles on many of the points above.

Software and code

Policy information about availability of computer code

Data collection	No software was used for data collection.
Data analysis	The data was mainly analysed with INSurVeyor: https://github.com/kensung-lab/INSurVeyor. GEMMA 0.98.1 and admixture 1.3.0 were used for the GWAS study, RepeatMasker 4.1.2-p1 for repeat annotation. Delly 0.9.1, Manta 1.6.0, MELT 2.2.2, xTEA (cloned from https://github.com/parklab/xTea in Dec 2021), Pamir 2.1.0, Sniffles2 2.0.7 were run to provide a baseline for comparison. BWA MEM 0.7.17 and samtools 1.14 were used to generate the BAM file for HG002. blastn was used to map the Arabidopsis insertions to the TE library. SVComparator (https://github.com/Mesh89/SVComparator) was used to benchmark the insertions.

For manuscripts utilizing custom algorithms or software that are central to the research but not yet described in published literature, software must be made available to editors and reviewers. We strongly encourage code deposition in a community repository (e.g. GitHub). See the Nature Portfolio guidelines for submitting code & software for further information.

Data

Policy information about availability of data

All manuscripts must include a data availability statement. This statement should provide the following information, where applicable:

- Accession codes, unique identifiers, or web links for publicly available datasets
- A description of any restrictions on data availability
- For clinical datasets or third party data, please ensure that the statement adheres to our policy

The insertions data generated in this study have been deposited in EBI. The insertions calls from 3,202 samples from the 1000 Genomes Project are under project PRJEB59423. The clustered calls are available under analysis ERZ16007666, while the single-sample calls are available under analysis ERZ16007665. The insertion calls from 1,047 Arabidopsis Thaliana from the 10001 Genomes Project are under project PRJEB58052. The clustered calls are available under analysis ERZ14864777, while the single-sample calls are available under analysis ERZ16031661. Sequencing data for HG002 was downloaded from NCBI (accessions SRR1766442 to SRR1766486). PacBio HiFi data for HG002 was downloaded from NCBI (PRJNA586863).
The HG002 benchmark catalogue and the list of tier 1 regions were downloaded from ftp://ftp-trace.ncbi.nlm.nih.gov/ReferenceSamples/giab/ data/ AshkenazimTrio/analysis/NIST_SVs_Integration_v0.6/. Information on accessing the 3,202 CRAM files for the 1KGP project produced by NYGC can be found at https://www.internationalgenome. org/data-portal/data-collection/30x-grch38. The Phase 2 benchmark calls produced by HGSVC are available at https:// www.internationalgenome. org/data-portal/data-collection/hgsvc2. The SV catalogue produced by NYGC was downloaded from http://ftp.1000genomes.ebi.ac.uk/ vol1/ftp/data_collections/1000G_2504_high_coverage/working/20210124. SV_Illumina_Integration/1KGP_3202.gatksv_svtools_novelins.freeze_ V3.wAF.vcf.gz. RepeatMasker annotations for hg19 and hg38 were down- loaded from the UCSC Table Browser.
Data from the 1001 Genomes Project was downloaded from NCBI (PRJNA273563). The TE library of Arabidopsis Thaliana was downloaded from https:// arabidopsis.org/ download_files/Genes/TAIR10_genome_release/. Phenotypic data was downloaded from the Arapheno database.

Human research participants

Policy information about studies involving human research participants and Sex and Gender in Research.

Reporting on sex and gender	Neither sex nor gender were considered in the study.
Population characteristics	The only population characteristics considered was ethnicity, both in the form of superpopulation (East Asian, European, African, Central/South American, South Asian) and subpopulation.
Recruitment	We used publicly available data.
Ethics oversight	We used publicly available data.

Note that full information on the approval of the study protocol must also be provided in the manuscript.

Field-specific reporting

Please select the one below that is the best fit for your research. If you are not sure, read the appropriate sections before making your selection.

☒ Life sciences ☐ Behavioural & social sciences ☐ Ecological, evolutionary & environmental sciences

For a reference copy of the document with all sections, see nature.com/documents/nr-reporting-summary-flat.pdf

Life sciences study design

All studies must disclose on these points even when the disclosure is negative.

Sample size	We aimed at demonstrating the performance of our caller on both human and non-human data. For this reason, we used two datasets: the full 1000 Genome Project, and the 1047 A.Thaliana samples from the 1001 Genome Project used in [https://doi.org/10.1093/nar/gkab904].
Data exclusions	No data was excluded.
Replication	We ensured that running the software on the same sample multiple times would produced consistent results.
Randomization	Randomization was not applicable to any of the analysis or experiments performed. For the GWAS analysis, allocation is performed based on presence/absence of the tested insertion.
Blinding	Blinding was not applicable to any of the analysis or experiments performed.

Reporting for specific materials, systems and methods

We require information from authors about some types of materials, experimental systems and methods used in many studies. Here, indicate whether each material, system or method listed is relevant to your study. If you are not sure if a list item applies to your research, read the appropriate section before selecting a response.

Materials & experimental systems

n/a	Involved in the study
☒	☐ Antibodies
☒	☐ Eukaryotic cell lines
☒	☐ Palaeontology and archaeology
☒	☐ Animals and other organisms
☒	☐ Clinical data
☒	☐ Dual use research of concern

Methods

n/a	Involved in the study
☒	☐ ChIP-seq
☒	☐ Flow cytometry
☒	☐ MRI-based neuroimaging
